# Author Correction: Smac mimetics and oncolytic viruses synergize in driving anticancer T-cell responses through complementary mechanisms

**DOI:** 10.1038/s41467-018-04597-8

**Published:** 2018-05-24

**Authors:** Dae-Sun Kim, Himika Dastidar, Chunfen Zhang, Franz J. Zemp, Keith Lau, Matthias Ernst, Andrea Rakic, Saif Sikdar, Jahanara Rajwani, Victor Naumenko, Dale R. Balce, Ben W. Ewanchuk, Pankaj Tailor, Robin M. Yates, Craig Jenne, Chris Gafuik, Douglas J. Mahoney

**Affiliations:** 1 Alberta Children’s Hospital Research Institute, Calgary, AB Canada T2N 4N1; 2 Arnie Charbonneau Cancer Institute, Calgary, AB Canada T2N 4N1; 3 Department of Microbiology, Immunology and Infectious Disease, Faculty of Medicine, University of Calgary, Calgary, AB Canada T2N 4N1; 4 Snyder Institute for Chronic Disease, Calgary, AB Canada T2N 4N1; 5 Department of Medical Sciences, Faculty of Medicine, University of Calgary, Calgary, AB Canada T2N 4N1; 6 Department of Comparative Biology and Experimental Medicine, Faculty of Veterinary Medicine, University of Calgary, Calgary, Canada T2N 4N1; 7 Department of Biochemistry and Molecular Biology, Faculty of Medicine, University of Calgary, Calgary, AB Canada T2N 4N1

Correction to: *Nature Communications*. 10.1038/s41467-017-00324-x; published online 24 August 2017

The originally published version of this article contained an error in the spelling of the author Pankaj Tailor, which was incorrectly given as Pankaj Taylor. This has now been corrected in both the PDF and HTML versions of the article.

