# Supplementary material for: Smac mimetics and oncolytic viruses synergize in driving anticancer T-cell responses through complementary mechanisms
Source: Nat Commun. 2017 Aug 24;8:344. doi: 10.1038/s41467-017-00324-x (PMC5570934; doi:10.1038/s41467-017-00324-x)
Supplement: Supplementary file 1 — Supplementary Information [file 41467_2017_324_MOESM1_ESM.pdf]

Description of Supplementary Files

File Name: Supplementary Information
Description: Supplementary Figures and Supplementary Table

File Name: Peer Review File
Description:

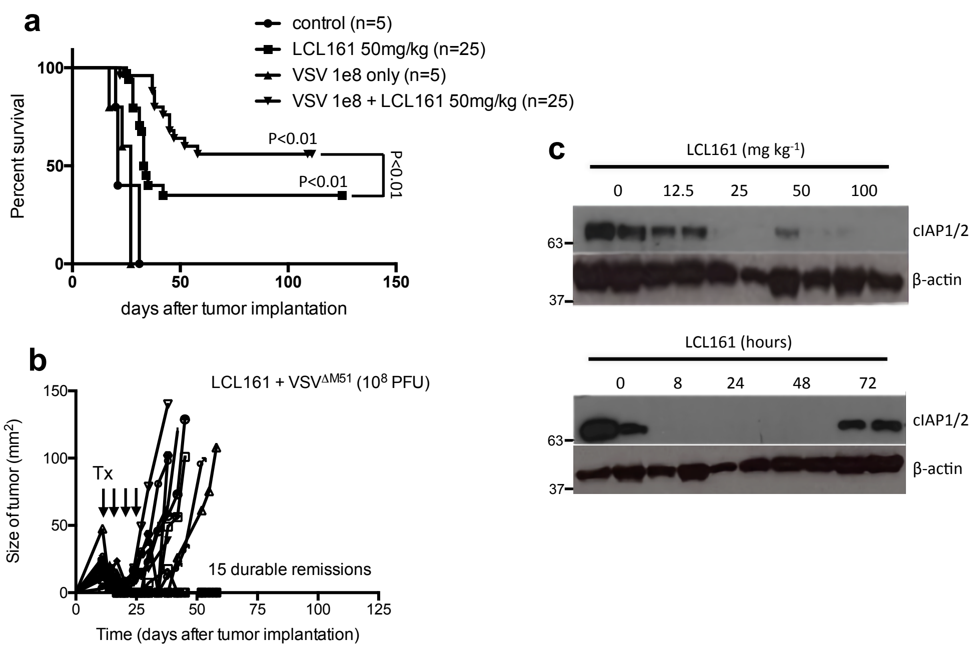

Supplementary Figure 1. LCL161±VSV$^{\Delta M51}$ therapy can elicit complete and durable EMT6 tumour control in Balb/c mice. **(a)** Overall survival of EMT6 tumour-bearing mice in response to the indicated treatments (triplicate experiments; log-rank test). **(b)** EMT6 tumour growth rate in response to LCL161+VSV$^{\Delta M51}$ combination therapy, measured by skin calipers. **(c)** cIAP1/2 protein in EMT6 tumours extracted from mice treated with LCL161, detected by immunoblots (n=2 biological replicates per experiment, duplicate experiments).

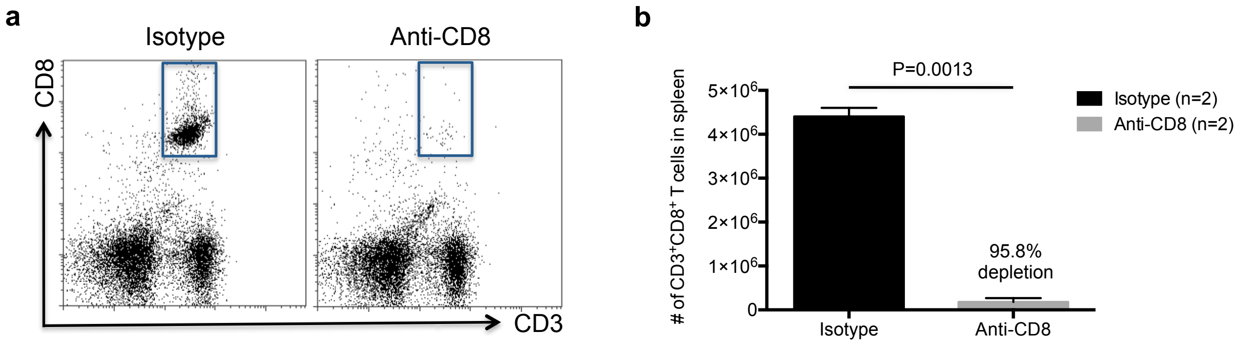

Supplementary Figure 2. Flow cytometry for $CD8^+$ T cells following neutralizing antibody treatment. **(a)** Representative flow cytometry dot plots of splenic $CD3^+/CD8^+$ T cells. **(b)** Splenic $CD3^+CD8^+$ T cells 3 days after αCD8 (or isotype) antibody treatment, measured by flow cytometry (duplicate experiments; mean±SD; *t*-test).

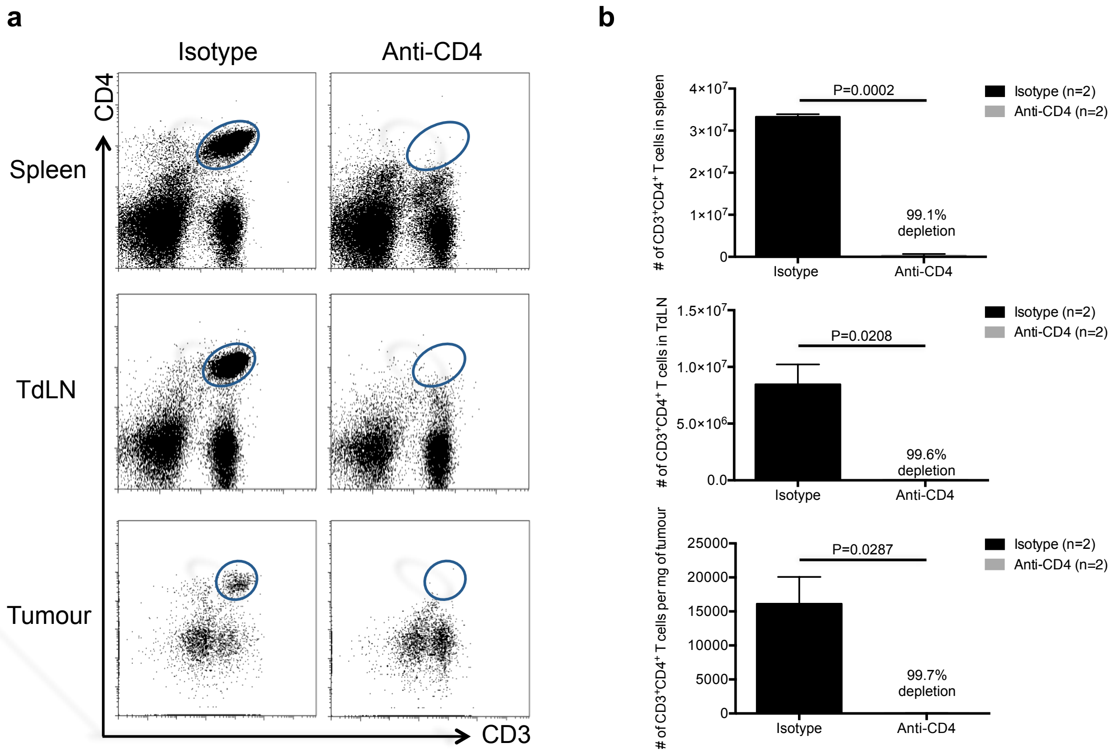

Supplementary Figure 3. Flow cytometry for $CD4^+$ T cells following neutralizing antibody treatment. **(a-c)** Representative flow cytometry dot plots of $CD3^+CD4^+$ T cells in spleen (a), TdLN (b) and EMT6 tumour (c). **(d-f)** Splenic $CD3^+CD4^+$ T cells in spleen (d), TdLN (e) and EMT6 tumour (f) 3 days after αCD4 (or isotype) antibody treatment initiation, measured by flow cytometry (single experiment; mean±SD; *t*-test).

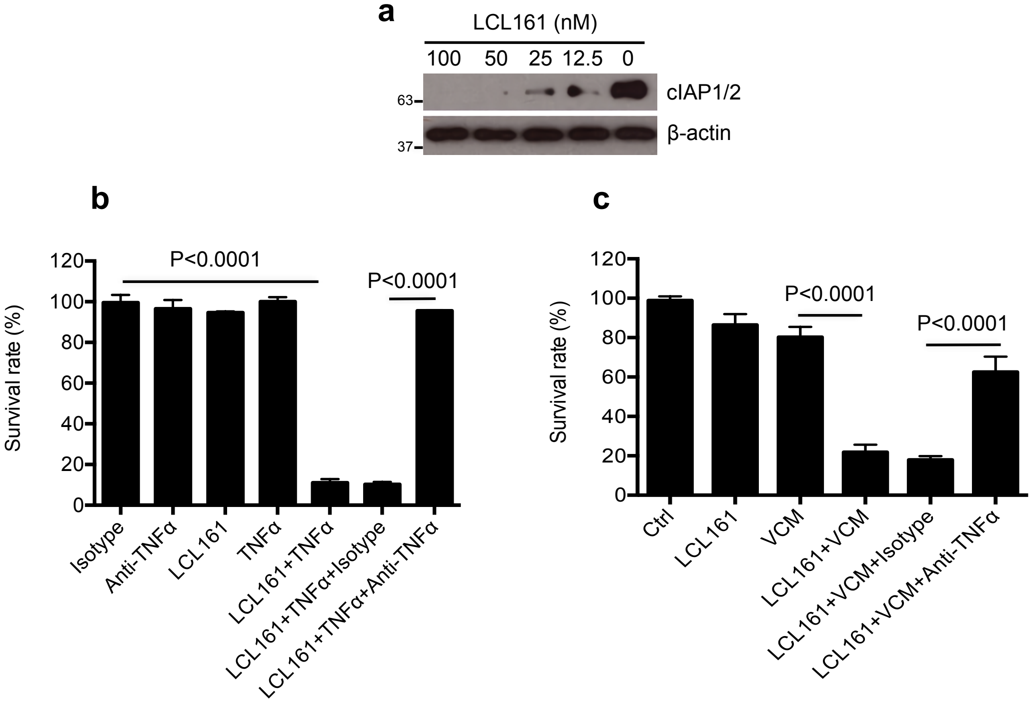

Supplementary Figure 4. LCL161 treatment leads to cIAP1/2 depletion and sensitizes EMT6 cells to TNFα-mediated cell death. **(a)** cIAP1/2 protein in EMT6 cells after treatment with LCL161 for 2 hours, detected by immunoblots (n=2 biological replicates, duplicate experiments). **(b)** EMT6 cell viability 48 hours after LCL161±recombinant TNFα (100 ng mL^{-1}) ± neutralizing antibody (TNFα or control), measured by Alamar Blue (n=3 biological replicates, duplicate experiments; mean±SD; ANOVA with Tukey's multiple comparisons test). **(c)** EMT6 cell viability 48 hours after LCL161±virus-conditioned media (VCM)±neutralizing antibody (TNFα or control), measured by Alamar Blue (n=3 biological replicates per experiment, duplicate experiments; mean±SD; ANOVA with Tukey's multiple comparisons test).

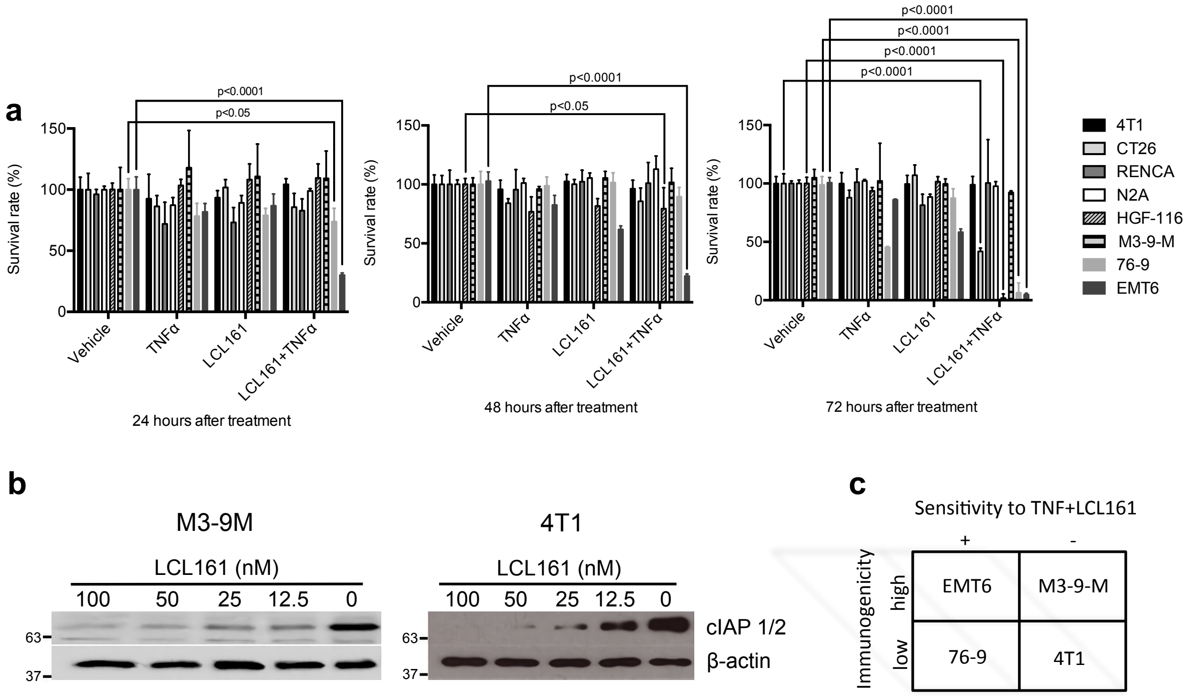

Supplementary Figure 5. Most mouse cancer cell lines are not sensitized to TNFα-mediated death by LCL161. **(a)** Cell viability 24, 48 and 72 hours after LCL161+recombinant TNFα treatment, measured by Alamar Blue (n=3 biological replicates per experiment, duplicate experiments; mean±SD; ANOVA with Tukey's multiple comparisons test). **(b)** cIAP1/2 protein in M3-9-M and 4T1 cells treated with LCL161 for 2 hours, detected by immunoblots (n=2 biological replicates per experiment, single experiment). **(c)** Tabular representation of sensitivity to LCL161+ TNFα versus immunogenicity for the mouse cancer cell lines chosen for analysis *in vivo*.

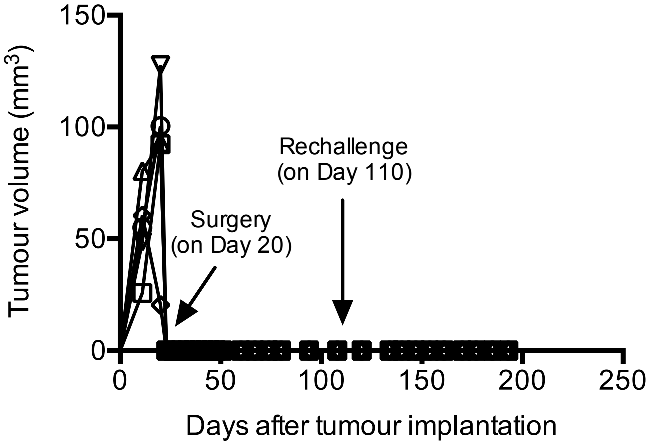

Supplementary Figure 6. EMT6 tumours are highly immunogenic. 12 day old EMT6 tumour-bearing mice underwent surgical resection and, 90 days later, rechallenged with 1e5 EMT6 cells. Tumour growth was monitored and plotted (single experiment).

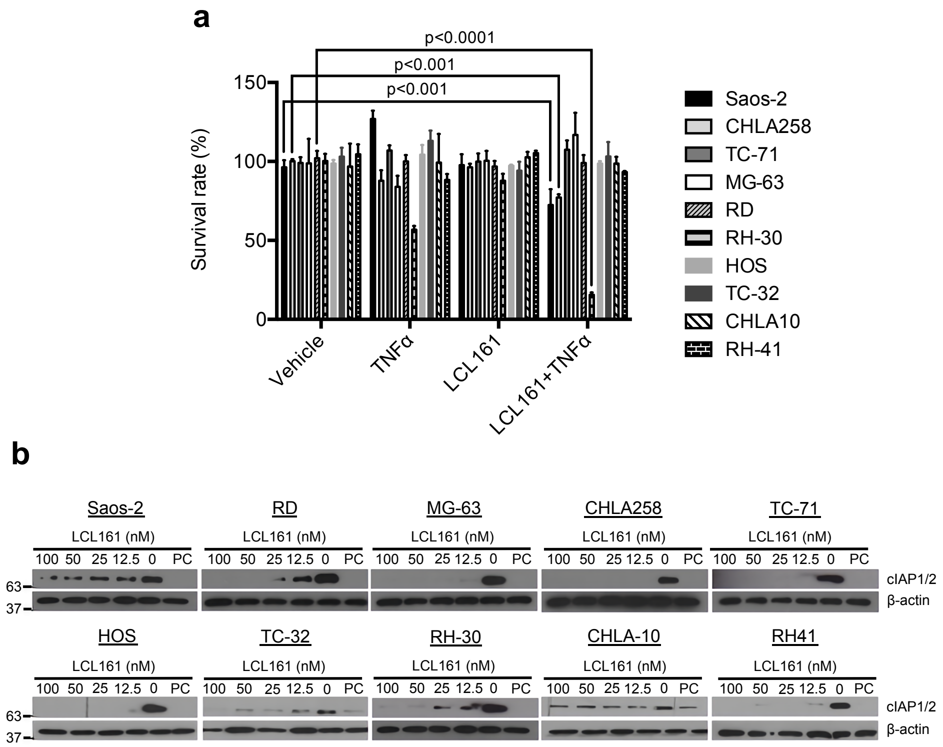

Supplementary Figure 7. Most human pediatric sarcoma lines are not sensitized to TNF α -mediated death by LCL161. **(a)** Viability of human pediatric sarcoma cells 48 hours after LCL161±recombinant TNFα treatment, measured by Alamar Blue (n=3 biological replicates per experiment, duplicate experiments; mean±SD; ANOVA with Tukey's multiple comparisons test). **(b)** cIAP1/2 protein in human pediatric sarcoma cells treated with LCL161 for 2 hours, detected by immunoblots (n=2 biological replicates, single experiment).

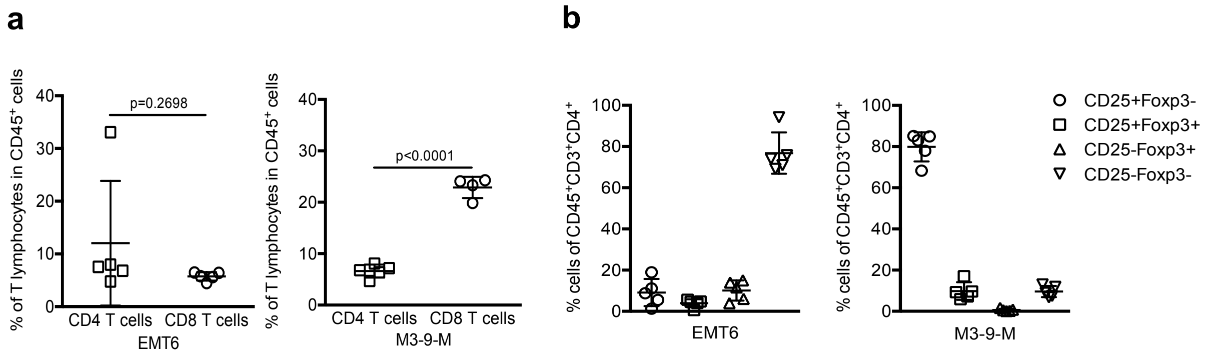

Supplementary Figure 8. Divergent CD4$^+$ polarization in EMT6 vs. M3-9-M tumours. **(a)** Total number of CD8$^+$ and CD4$^+$ T cells infiltrating 12 day old EMT6 or M3-9-M tumours, measured by flow cytometry (duplicate experiments; mean±SD; *t*-test). **(b)** Relative number of CD4$^+$ T cells expressing CD25 and/or Foxp3 polarization markers (duplicate experiment; mean±SD; ANOVA with Tukey's multiple comparisons test).

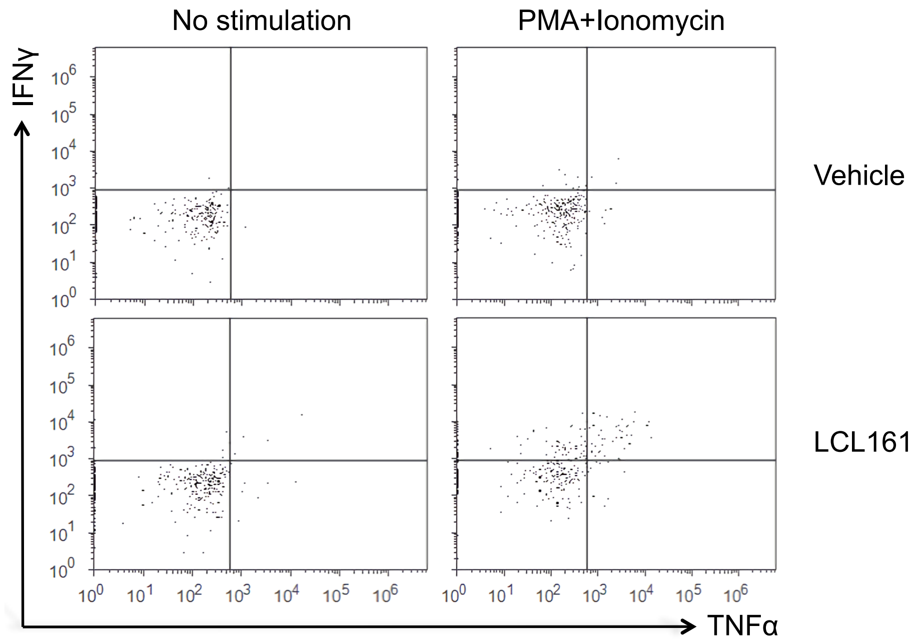

Supplementary Figure 9. Flow cytometry for intracellular cytokine staining in tumour-infiltrating $CD8^+$ T cells. Representative dot plots of IFNγ- or TNFα- or IFNγ/TNFα- positive cells within the $CD45^+CD3^+CD8^+$ leukocyte fraction isolated from EMT6 tumours.

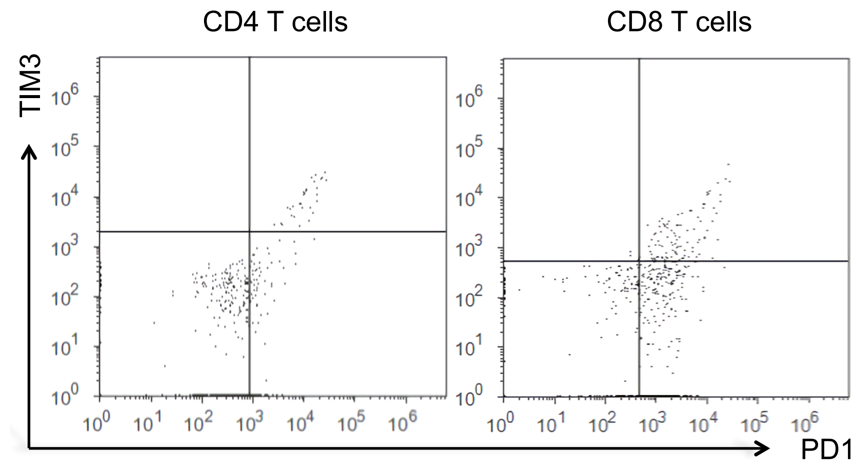

Supplementary Figure 10. Representative dot plots of PD-1 or Tim-3 or PD-1/Tim-3- positive cells within the $CD45^{+}CD3^{+}CD8^{+}$ leukocyte fraction isolated from EMT6 tumours.

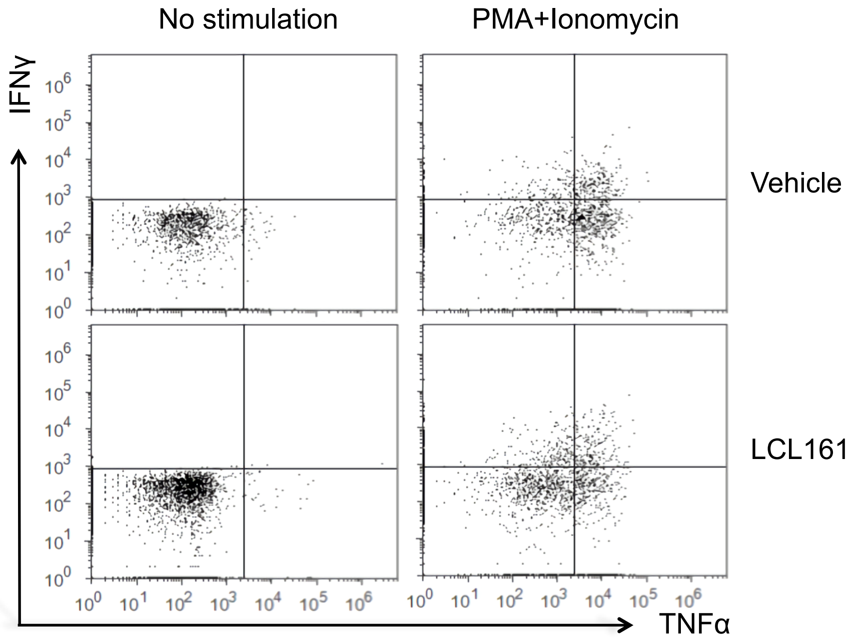

Supplementary Figure 11. Flow cytometry for intracellular cytokine staining in TdLN $CD8^+$ T cells. Representative dot plots of IFNγ- or TNFα- or IFNγ/TNFα- positive cells within the $CD45^+CD3^+CD8^+$ fraction isolated from EMT6 TdLN.

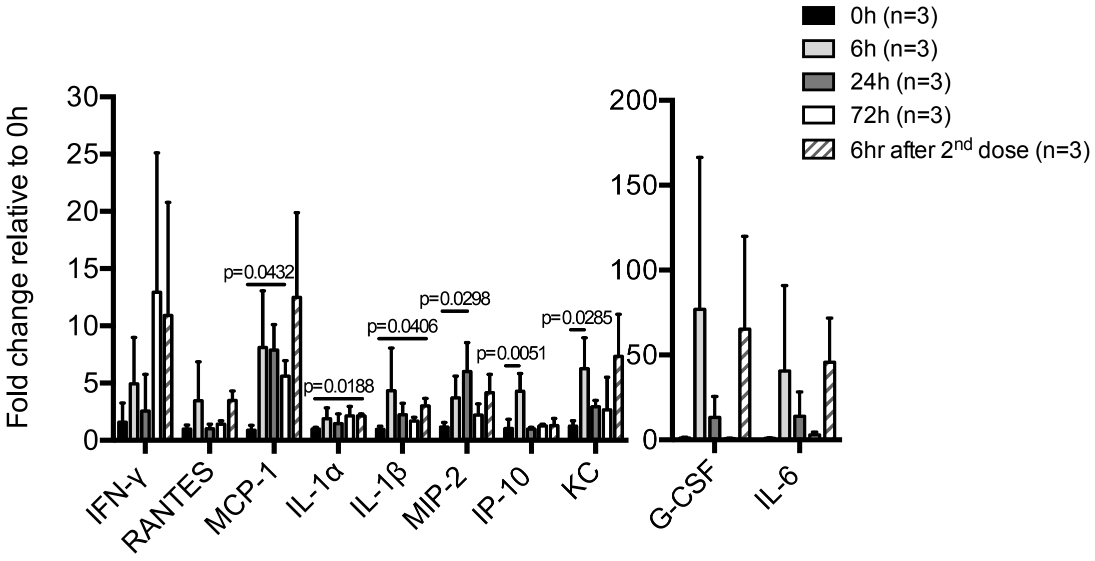

Supplementary Figure 12. LCL161 alters the cytokine milieu within EMT6$^{TNFR1-/-}$ tumours (clone 2-10) toward an immunostimulatory cytokine signature. Cytokine expression within the interstitial fluid of EMT6 tumours, measured by Luminex (n=3 biological replicates; single experiment; mean±SD; ANOVA with Tukey's multiple comparisons test).

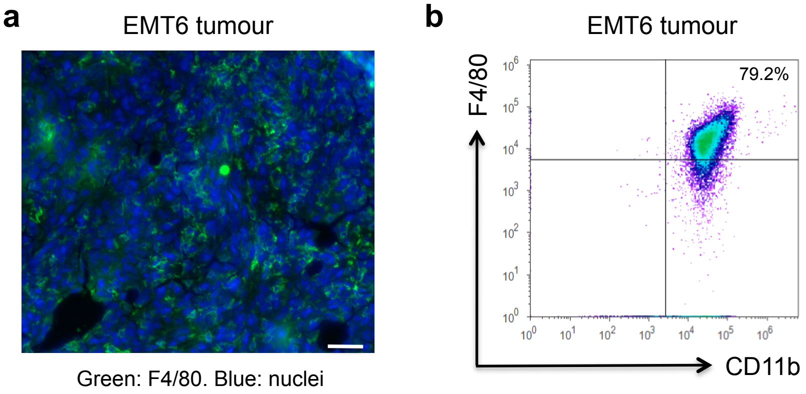

Supplementary Figure 13. EMT6 tumours are highly enriched with macrophages. **(a)** Representative immunofluorescence staining of F4/80^{+} macrophages in EMT6 tumours. Green: F4/80. Blue: nuclei (DAPI). Scale bar=50μM. **(b)** Representative flow cytometry dot plot for CD11b^{+}F4/80^{+} cells within the CD45^{+} fraction isolated from EMT6 tumours (n=4 biological replicates per experiment, duplicate experiments).

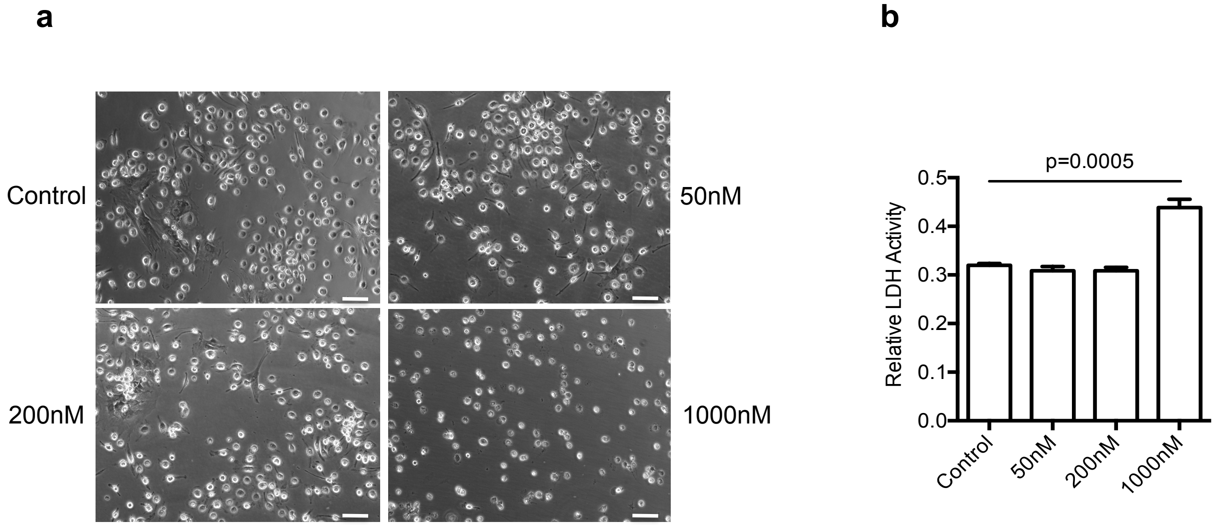

Supplementary Figure 14. LCL161 treatment reduces BMDM viability. **(a)** Representative brightfield images of cultured BMDM treated with LCL161 for 48 hours. Scale bar=50μM. **(b)** LDH activity in BMDMs treated with LCL161 for 48 hours at the indicated concentrations (n=3 biological replicates, duplicate experiments; mean±SD; ANOVA with Tukey's multiple comparisons test).

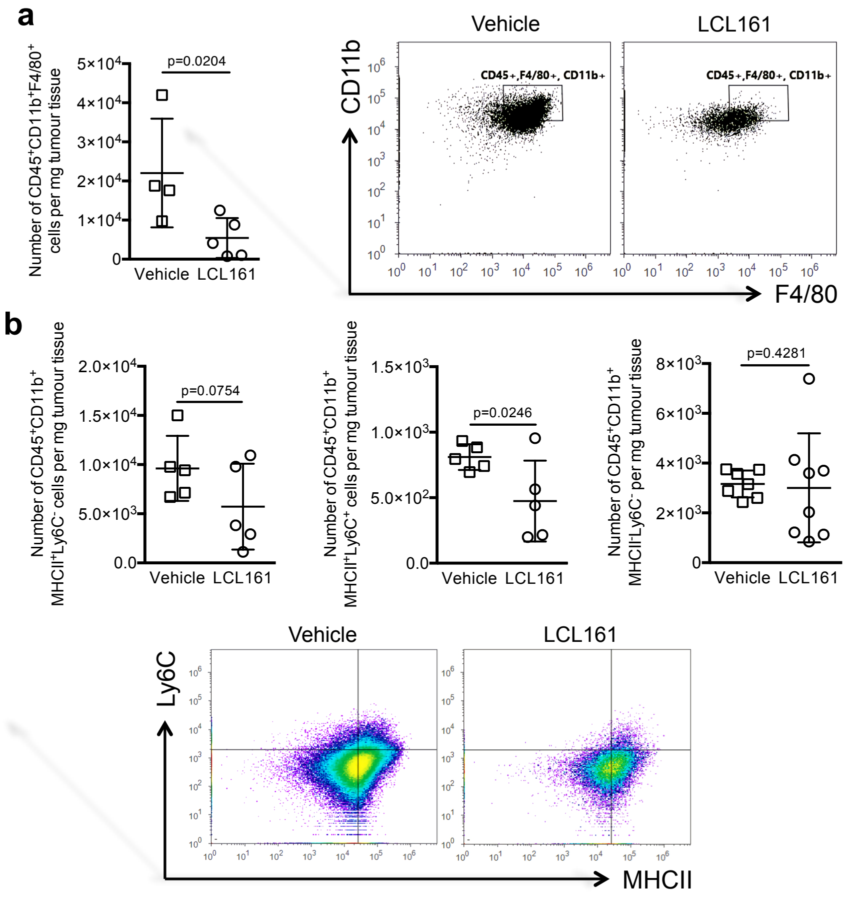

Supplementary Figure 15. LCL161 treatment reduces TAMs in EMT6 tumours. **(a)** ***Left panel***: Total number of $CD45^{+}CD11b^{+}F4/80^{+}$ in EMT6 tumours 72 hours after LCL161 (or vehicle) treatment, measured by flow cytometry (triplicate experiments; mean±SD; *t*-test). ***Right panel***: Representative dot plots. **(b)** ***Top panel:*** Total number of $CD45^{+}CD11b^{+}MHC\text{-}II^{\pm}Ly6C^{\pm}$ in EMT6 tumours 72 hours after LCL161 (or vehicle) treatment, measured by flow cytometry (triplicate experiments; mean±SD; *t*-test). ***Bottom panel***: Representative dot plots.

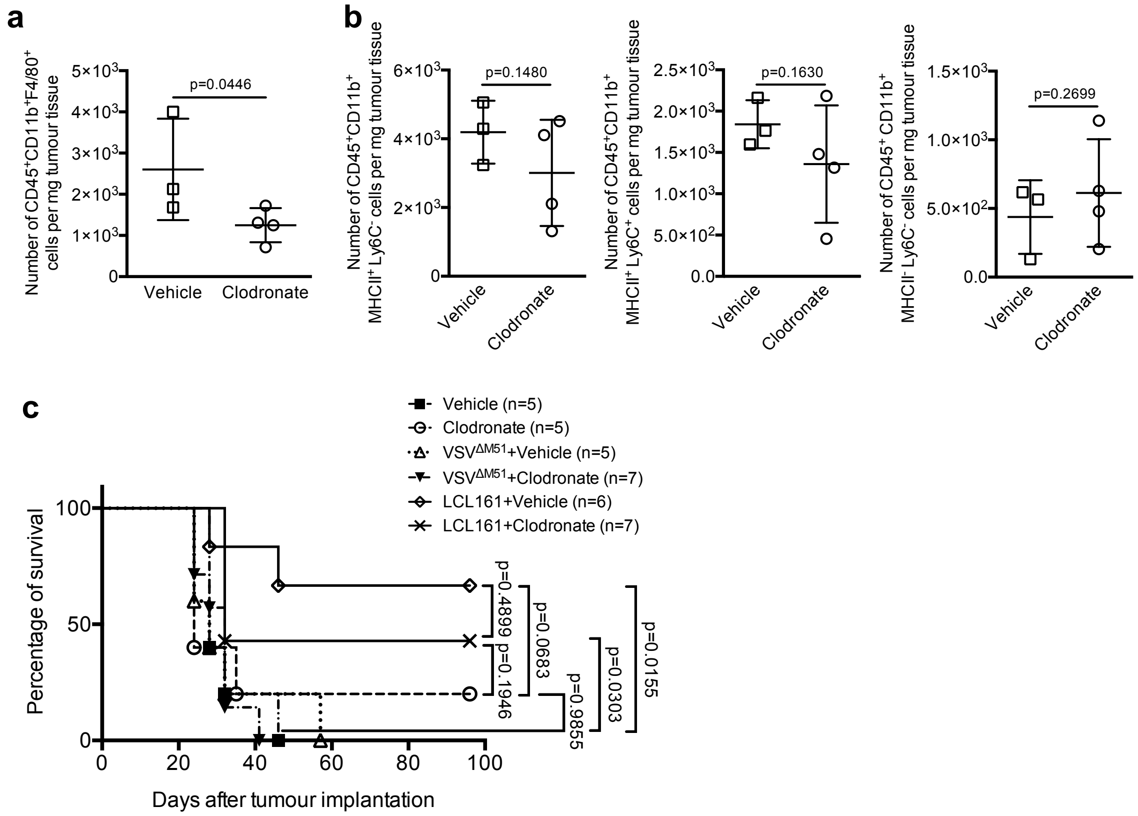

Supplementary Figure 16. Clodronate liposome-mediated TAM depletion does not phenocopy LCL161 therapy. **(a)** Total number of CD45$^+$CD11b$^+$F4/80$^+$ in EMT6 tumours 7 days after CL (or vehicle) treatment, measured by flow cytometry (single experiment; mean±SD; *t*-test). **(b)** Total number of CD45$^+$CD11b$^+$MHC-II$^{\pm}$Ly6C$^{\pm}$ in EMT6 tumours 7 days after CL (or vehicle) treatment, measured by flow cytometry (single experiment; mean±SD; *t*-test). **(c)** Overall survival of EMT6 tumour bearing mice treated with the indicated therapies beginning on day 12. (single experiment; log-rank test)

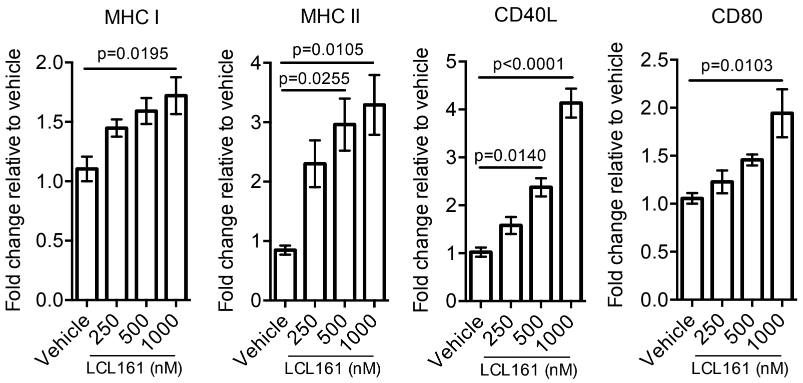

Supplementary Figure 17. LCL161 causes upregulation of MHC and co-stimulatory proteins on macrophages. BMDMs were treated with the indicated concentrations of LCL161 (or vehicle) for 20 hours prior to flow cytometry for MHC-I, MHC-II, CD40L and CD80 (n=3 biological replicates, duplicate experiments; mean±SD; ANOVA with Tukey's multiple comparisons test).

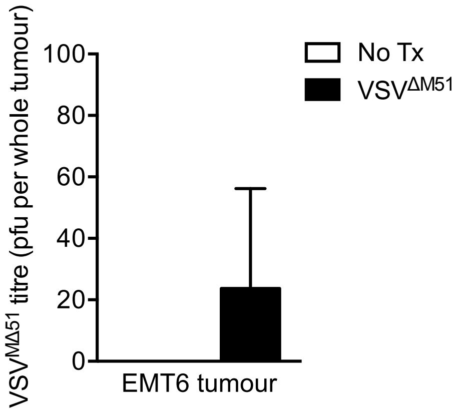

Supplementary Figure 18. Systemic delivery of $VSV^{\Delta M51}$ leads to a small infection of EMT6 tumours. 12 day old EMT6 tumour-bearing mice were treated with $VSV^{\Delta M51}$($1x10^8$ PFU) i.v. and 12 hours later infectious particles measured in tumours by plaque assay (n=3 biological replicates; single experiment; mean±SD).

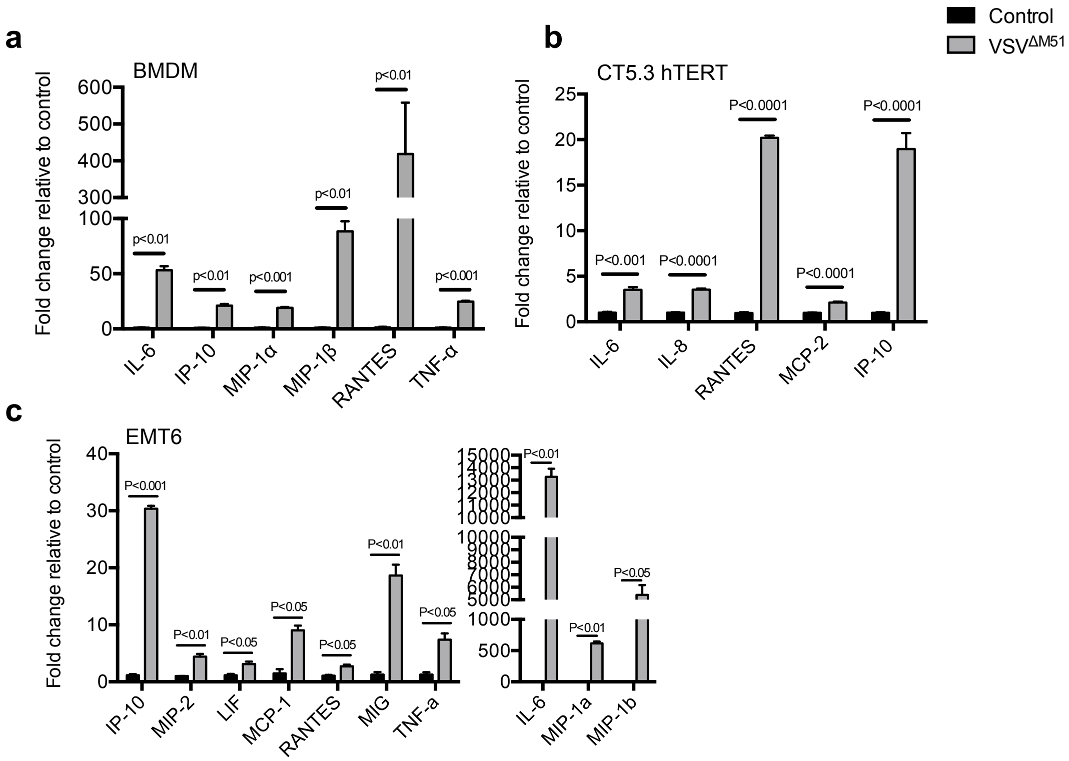

Supplementary Figure 19. VSV$^{\Delta M51}$ elicits cytokine secretion from cancer and normal cells *in vitro*. **(a-c)** Immunostimulatory cytokines measured from EMT6 (**a**, MOI=0.1, 48 hpi), BMDM (**b**, MOI=10, 24 hpi) and cancer-associated fibroblasts (CT5.3 hTERT, **c**, MOI=0.1, 48 hpi) culture after infection with VSV$^{\Delta M51}$ (n=3 biological replicates, single experiment; mean±SD; *t*-test).

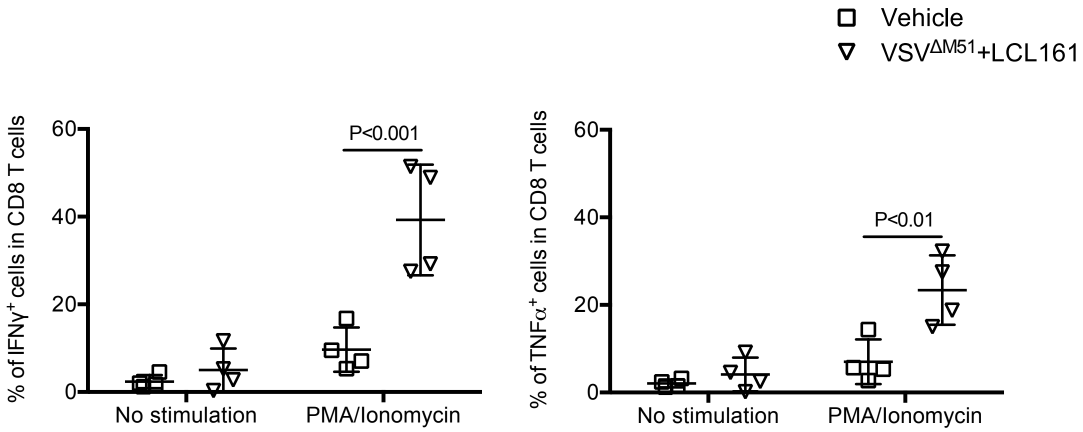

Supplementary Figure 20. LCL161 rejuvenates tumour-infiltrating T cells in mice bearing EMT6$^{TNFR1-/-}$ tumours. Intracellular staining for IFNγ or TNFα within CD8^{+} T cells isolated from EMT6$^{TNFR1-/-}$ tumours (clone 2-10) and stimulated with PMA and ionomycin *ex vivo*, measured by flow cytometry (single experiment; mean±SD; ANOVA with Bonferroni's multiple comparisons test).

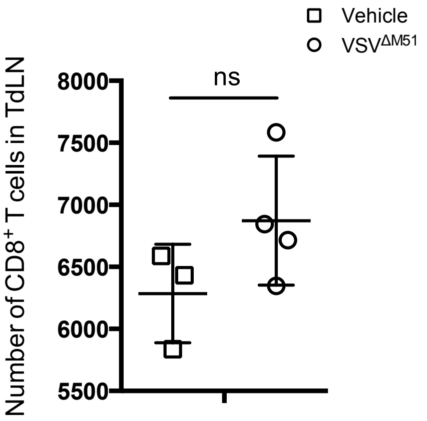

Supplementary Figure 21. VSV$^{\Delta M51}$ has no effect on the number of CD8^{+} T cells within the TdLN. CD8^{+} T cells within EMT6 TdLN 7 days after LCL161 (or vehicle) treatment initiation, measured by flow cytometry (duplicate experiments; mean±SD; *t*-test).

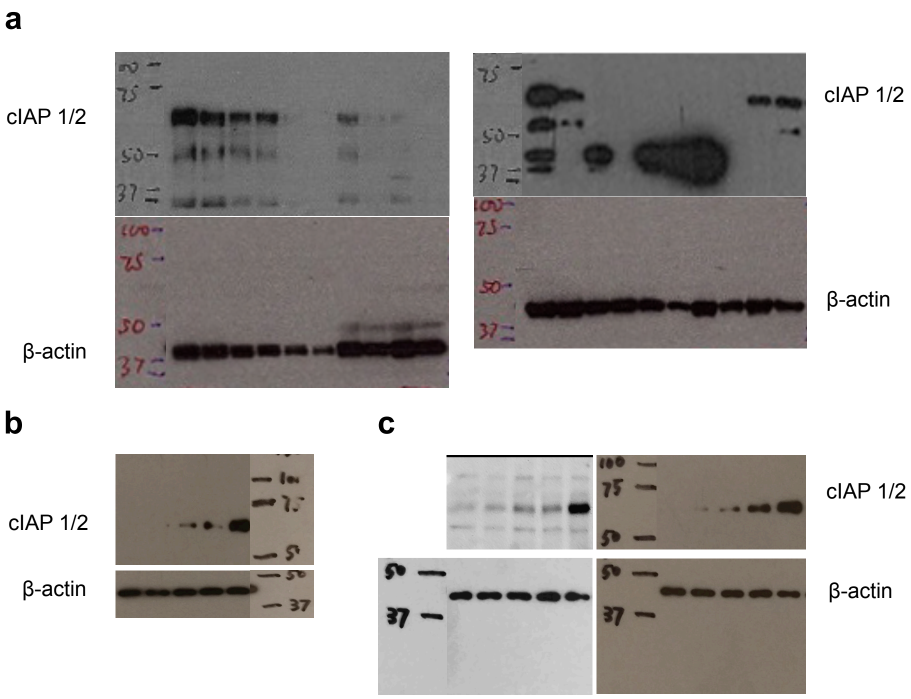

Supplementary Figure 22. Whole blots shown in (a) Supplementary Fig. 1c, (b) Supplementary Fig. 4a, (c) and Supplementary Fig. 5b

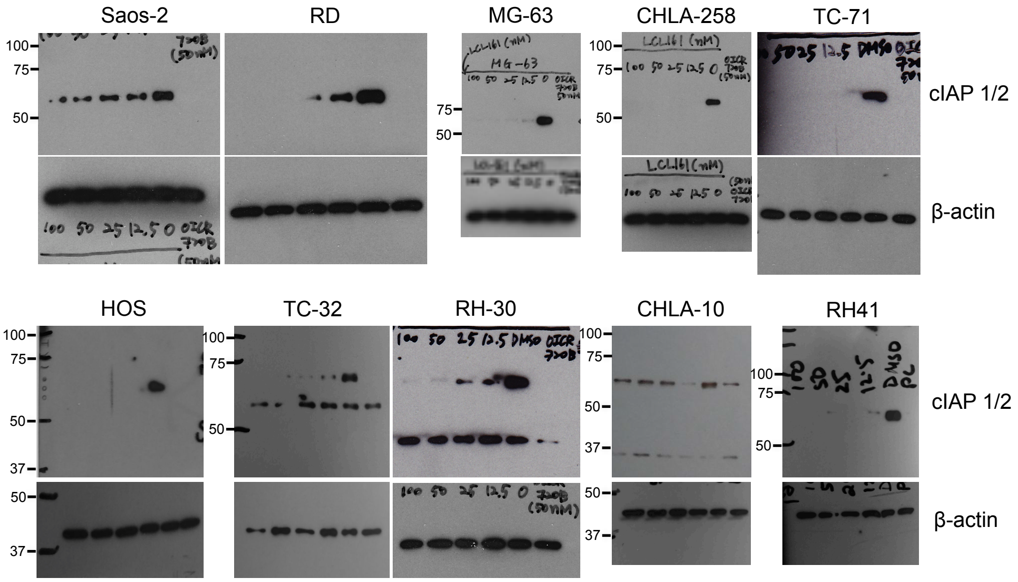

Supplementary Figure 23. Whole blots shown in Supplementary Fig. 7

Gene of Interest	Annealing Temp	Forward primer (5’-3’)	Reverse primer (5’-3’)
GAPDH	55.7	CACGGCAAATTCAACGGCACAGT	TGGGGGCATCGGCAGAAGG
IFN α	57	CTTCCACAGGATACTGTGTACCT	TTCTGCTCTGACCACCCTCCC
IFN β	57	ATGAGTGGTGGTTGCAGGC	TGACCTTTCAAATGCAGTAGATTCA
IRF7	59	AGCAAGACCGTGTTTACGAC	AGTGCTGAAGTCGAAGATGG
OAS	63.3	GCCATTGCACGCTCGCCTACTAC	CTCCTGCCATCCGGGTTTTTCA
STAT1	55	CTGAATATTTCCCTCCTGGG	TCCCGTACAGATGTCCATGAT
Mx2	57	CCTGCCTGCCATCGCTGTC	GCCTCTCCACTCCTCTCCCTCATT
iNOS	55	TTTGCTTCCATGCTAATGCGAAAG	GCTCTGTTGAGGTCTAAAGGCTCCG
Arg1	60.9	AGGGTTACGGCCGGTGGAGAG	CCCCTCCTCGAGGCTGTCCTTTT

Supplementary Table 1. Annealing temperatures and primer sequences used each gene specific semi-quantitative RT-PCR amplification
